# The Neglected Contribution of Streptomycin to the Tuberculosis Drug Resistance Problem

**DOI:** 10.3390/genes12122003

**Published:** 2021-12-17

**Authors:** Deisy M. G. C. Rocha, Miguel Viveiros, Margarida Saraiva, Nuno S. Osório

**Affiliations:** 1 Life and Health Sciences Research Institute (ICVS), School of Medicine, University of Minho, Campus Gualtar, 4710-057 Braga, Portugal; deisy.rocha@hotmail.com; 2 ICVS/3B’s, PT Government Associate Laboratory, 4710-057 Braga, Portugal; 3 i3S, Instituto de Investigacão e Inovação em Saúde, University of Porto, 4200-135 Porto, Portugal; margarida.saraiva@ibmc.up.pt; 4 IBMC, Instituto de Biologia Molecular e Celular, University of Porto, 4200-135 Porto, Portugal; 5 GHTM, Global Health and Tropical Medicine, Instituto de Higiene e Medicina Tropical da Universidade NOVA de Lisboa, 1349-008 Lisboa, Portugal; MViveiros@ihmt.unl.pt

**Keywords:** antibiotic, multidrug-resistance, drug-resistance, resistance level, mutations, lineages, *Mycobacterium*, streptomycin, tuberculosis

## Abstract

The airborne pathogen *Mycobacterium tuberculosis* is responsible for a present major public health problem worsened by the emergence of drug resistance. *M. tuberculosis* has acquired and developed streptomycin (STR) resistance mechanisms that have been maintained and transmitted in the population over the last decades. Indeed, STR resistant mutations are frequently identified across the main *M. tuberculosis* lineages that cause tuberculosis outbreaks worldwide. The spread of STR resistance is likely related to the low impact of the most frequent underlying mutations on the fitness of the bacteria. The withdrawal of STR from the first-line treatment of tuberculosis potentially lowered the importance of studying STR resistance. However, the prevalence of STR resistance remains very high, could be underestimated by current genotypic methods, and was found in outbreaks of multi-drug (MDR) and extensively drug (XDR) strains in different geographic regions. Therefore, the contribution of STR resistance to the problem of tuberculosis drug resistance should not be neglected. Here, we review the impact of STR resistance and detail well-known and novel candidate STR resistance mechanisms, genes, and mutations. In addition, we aim to provide insights into the possible role of STR resistance in the development of multi-drug resistant tuberculosis.

## 1. Introduction

Tuberculosis (TB) is an airborne transmissible infectious disease caused by the bacteria *Mycobacterium tuberculosis*. In 2020, over 10 million people developed TB, and more than 1.5 million died of it [1]. Eight countries account for two-thirds of the total number of new TB cases (India, China, Indonesia, the Philippines, Pakistan, Nigeria, Bangladesh, and South Africa). Drug resistance (DR) is pointed to as a major threat in the control of TB, likely impairing the goals established for 2030 by the World Health Organization (WHO) END TB strategy. In 2020, 132,222 rifampicin resistance/multi-drug resistant (RR/MDR) and 25,681 extensively drug resistant (XDR) or pre-XDR TB cases were reported [1]. Therefore, understanding the multitude of factors contributing to DR and MDR TB, and how to overcome them, is an urgency.

DR TB has been a problem for 73 years since the identification of the first streptomycin (STR)-resistant *M. tuberculosis* strains [2]. Starting from 1994, the Global Project on Anti-TB DR Surveillance has collected data on DR TB cases. The treatments of infections with drug-resistant *M. tuberculosis* strains are long and toxic; therefore, adherence is often poor, which favors DR. The problem then aggravates in a vicious cycle with the acquisition of more resistance mutations and transmission of the resistant strains [1]. The emergence of MDR and XDR strains has amplified the DR problem because these are sometimes highly transmissible and particularly hard to treat, hampering the success of treatment for up to 50% [3,4]. Until 2018, second-line antibiotics, such as fluoroquinolones, and injectable aminoglycosides, were recommended for 18 to 20 months to treat MDR and XDR infections. In 2019, the WHO published new guidelines replacing these long-term regimens for shorter oral bedaquiline-based regimens (9–12 months). In some cases, surgery may be used as adjunctive therapy for MDR or XDR TB patients [5,6,7,8,9]. Nevertheless, in cases where the new short oral–regimes cannot be applied, a recommended alternative is the administration of the injectable second-line drug STR [10,11]. 

## 2. Seventy Years of Streptomycin Resistance in Tuberculosis, Where Do We Stand?

STR is an antibiotic from the aminoglycoside group isolated from the actinomycete *Streptomyces griseus* [12]. It was described in 1944 as an efficient antibiotic against *M. tuberculosis* [13,14]. During the first 3 to 4 years after its discovery, STR was used as a monotherapy regimen to treat TB. However, with the emergence of resistant strains, a multi-therapy regimen was introduced, comprising STR in combination with INH and para-aminosalicylic acid (PAS), but once again, DR was reported [14,15]. STR was also part of the first short-course TB therapy, which combined this drug with three first-line antibiotics INH, RIF, and PZA. However, later, due to their debilitating side effects and resistance development when used as monotherapies, STR and PAS were substituted with another first-line antibiotic, ethambutol (ETH) [14,16,17]. From 1991, STR was only used in the so-called category II treatment, which consisted of the addition of STR to TB treatment after the failure of first-line therapy. Nowadays, category II treatment is not recommended, and STR has limited clinical application due to the high incidence of resistant strains. However, it is still in use in some cases as a substitute for amikacin against MDR-TB in the longer regimens or as an affordable alternative for low resource settings [10,13,18]. 

**Figure 1:**
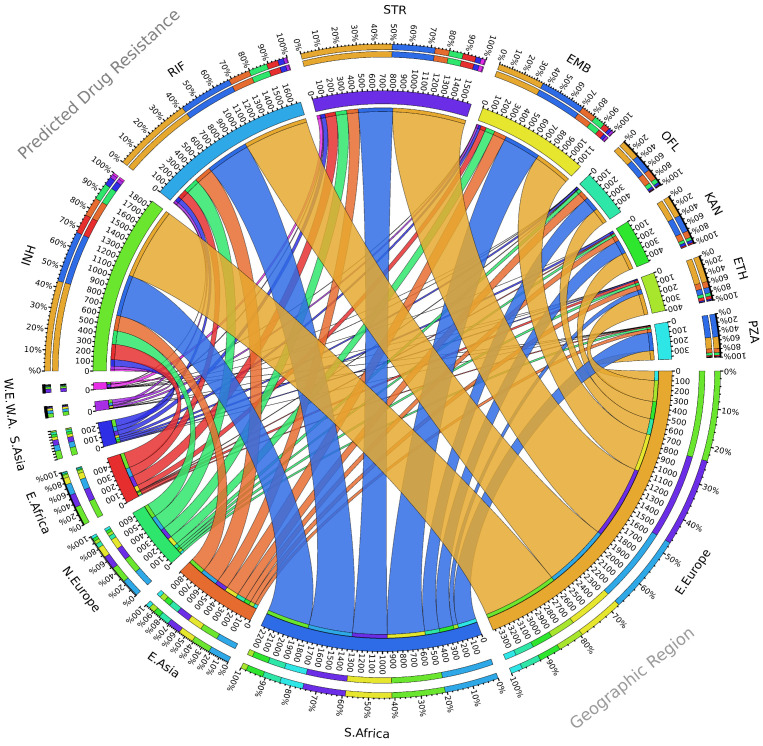
Circular visualization of data from Manson et al. [19] regarding the computationally predicted drug resistance profile of the *M. tuberculosis* clinical isolates from the global regions most represented in the study [19]. The number of analyzed genomes is represented in the inner ring and the relative percentage in the outer ring. The connection between predicted drug resistance and geographic region is shown by colored ribbons. Abbreviations: isoniazid (INH), rifampicin (RIF), streptomycin (STR), ethambutol (EMB), ofloxacin (OFL), kanamycin (KAN), ethionamide (ETH), Pyrazinamide (PZA), Eastern Europe (E. Europe), Southern Africa (S. Africa), Eastern Asia (E. Asia), Northern Europe (N. Europe), Eastern Africa (E. Africa), Southern Asia (S. Asia), Western Africa (W. A.), Western Europe (W. E.).

Although STR is currently used less in TB treatment, STR-resistant strains are still common among different *M. tuberculosis* lineages spread worldwide and still frequent in different parts of the globe (Figure 1), including Europe [19]. In Germany, monoresistance to STR was the most prevalent form of DR, and also, the most frequent resistance among MDR strains [20]. In Portugal, mutations in STR target genes were found in isolates from the MDR Lisbon family genetic cluster Q1, being, furthermore, considered as a surrogate marker for Q1 isolates [21,22]. Moreover, between 2007 and 2013, STR resistance accounted for 82.7% of DR TB cases diagnosed in a pulmonary TB cohort from Porto, north of Portugal [23,24,25]. In a retrospective study with patients from China, STR resistance was found at high frequency, present in 64.9% of the cases, and it was also highly prevalent in Mexico, Iran, and other endemic TB regions [26,27,28,29]. Different studies with drug-resistant *M. tuberculosis* clinical isolates from Myanmar and Thailand, two TB high burden countries, also reported a high incidence of STR resistance [30,31,32]. Moreover, considering the incomplete knowledge of all the mutations conferring STR resistance, it is likely that the prevalence of STR resistance is being underreported, as several studies are based solely on genotypic data. STR resistance is widely spread, even though it is, nowadays, less frequently used to treat TB. Although the exact reason for the high incidence of STR resistance is not well established, it is likely a long-term consequence of its use as a monotherapy decades ago, before the introduction of combined therapy with more effective antibiotics, like RIF and ETH [33,34,35].

Most importantly, STR resistance is also frequent in settings with a high MDR and XDR TB burden, with a series of studies highlighting its possible contribution to the development of MDR/XDR TB. A whole genome-based study placed STR resistance as one of the precursors of MDR in TB [19]. In another study, this fact was also highlighted because it was found that the order of resistance acquisition was first isoniazid resistance, followed by rifampicin and streptomycin, and only then resistance to other drugs [36]. Additionally, in Vietnam, STR resistance was pointed as a possible prerequisite for “Beijing strains” to transmit and develop MDR [37,38]. Resistant mutations in the three STR target genes were also associated with MDR in studies conducted in Poland and Zambia [39,40]. Furthermore, in the most lethal XDR TB outbreak of an epidemic clone in South Africa, which started in 2005, the first step towards XDR-level drug resistance was the acquisition of INH and STR resistance, acquired 50 years prior to the Tugela Ferry outbreak [41]. Understanding how STR resistance is connected to the development of MDR/XDR TB is fundamental.

All in all, STR resistance may still subsist as a clinically relevant problem, calling for more studies in the field.

## 3. Emergence of Streptomycin-Resistant Strains

### 3.1. Mode of Action of Streptomycin

**Figure 2:**
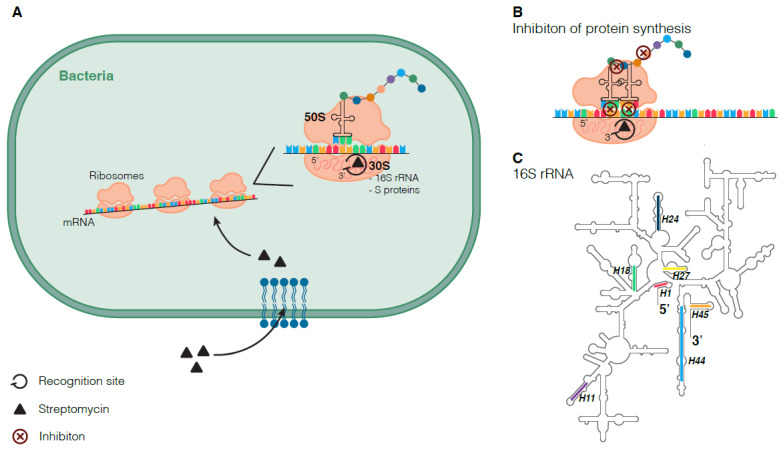
Inhibition of protein synthesis through the interaction of STR and 30S ribosome subunit. Schematic representation on STR entry in the cell and binding of STR to the 30S ribosomal subunit (**A**). Schematic representation of the inhibition of protein synthesis after the interaction of STR with the 30S subunit (**B**). Secondary structure of 16S rRNA highlighting relevant helices (**C**).

Like other members of the aminoglycoside group, STR interacts with bacterial cell surfaces through ionic bonding to access the periplasmic space [13,42]. It is then transported into the cytoplasm by membrane channels created by proton motive forces, like electron transporters. Once inside the cytoplasm, STR binds to the 30S ribosome subunit [13,42]. This binding occurs upon the establishment of chemical interactions between the drug, different regions in the 16S rRNA, and the K45 residue in the S12 ribosome protein (Figure 2) [42,43,44]. These interactions block elongation, inhibit initiation, and promote the misreading of codons, thus hampering protein synthesis and translational proofreading and ensuring the bactericidal effect [42,44]. Nonetheless, aminoglycosides are known to be less active under anaerobic conditions, such as the ones commonly found within the granulomas of TB patients [42,44].

### 3.2. Molecular Mechanisms of Streptomycin Resistance

#### 3.2.1. Streptomycin Resistance Mutations in *rpsL*, *rrs*, and *gid*

Resistance to STR is primarily associated with mutations in the *M. tuberculosis* genome occurring in the genes *rpsL*, *rrs*, and *gid*. These genes encode for the S12 protein, 16S rRNA, and the S-adenosyl methionine dependent 7-methylguanosine methyltransferase, respectively [28,39,45,46,47,48,49,50,51]. The most-reported STR resistant mutations occur in codons 43 and 88 of the *rpsL* gene or in the surroundings of nucleotides 530 and 912 of *rrs.* The mutation Lys43Arg in *rpsL* was frequently found among the so-called “Beijing strains”, a lineage 2 genotype often associated with MDR and XDR outbreaks. Several STR resistant variants commonly identified in *gid* are non-synonymous SNPs. Furthermore, a frameshift mutation in this locus can also contribute to the development of STR resistance [21,32,52,53,54]. In a recent study, novel candidates of STR resistance mutations not listed in the major DR mutation databases were identified [23]. Interestingly three of them, *gid* (Ile11Thr, Cys191Trp, and Val112Gly), were identified in inferred transmission clusters containing only isolates that were phenotypically resistant to STR, suggesting high transmissibility [23]. Furthermore, another recent study also proposed that the substitution of Val for Gly in the amino acid 112 of *gid* was associated with STR resistance [55]. The incomplete knowledge on all mutations that confer STR resistance is likely causing underreporting of the DR levels in studies only based on genetic analysis. On the other hand, one variant in *gid,* Leu16Arg, was incorrectly described in the past as a resistant conferring mutation before different studies found that it was present both in STR susceptible and resistant clinical isolates from the *M. tuberculosis* LAM sub-lineage of lineage 4. Similarly, other STR polymorphisms were also described as markers for lineage 2 sub-lineages commonly known as “Beijing”. For example, the *gid* mutations Glu92Asp, Ala205Ala, and Val110Val were found regardless of the STR status in all clinical isolates from sub-lineages of lineage 2 [49,54,55,56,57,58]. Overall, these results suggest that the *gid* gene is likely involved in lineage or sub-lineage evolution in addition to its relevance in DR [21,49,51,55]. Similarly, the 491 c > t mutation in *rrs* was reported as a marker of the L4.3.2 (LAM3) genetic taxa within the LAM sub-lineage of lineage 4. Table 1 summarizes the mutations described in the literature to be associated with STR resistance in the genes *rpsL*, *rrs*, and *gid*. Of note, some of the indicated polymorphisms were only recently identified and associated with STR resistant *M. tuberculosis* isolates; it is possible that other mutations conferring STR resistance are still to be uncovered. 

It was previously suggested that different mutations in *rpsL*, *rrs*, and *gid* trigger different levels of STR resistance. For example, the polymorphism Lys43Arg in the *rpsL* gene was associated with high resistance levels (minimum inhibitory concentration (MIC) > 10 µg/mL), mutations in the *rrs* locus mapped in the 530 loop and the vicinity of the nucleotide 912 were associated with intermediate to high resistance levels, while several *gid* polymorphisms have been correlated with low-level STR resistance (MIC circa 1–2 µg/mL) [11,55,56,57]. Nevertheless, data from recent studies supported that mutations in *gid* can also be found in strains with high resistance levels [23,58]. It is likely that the different levels of STR resistance depend on the location of the mutation on *rpsL*, *rrs*, or *gid* genes, on the genomic context of the isolate, and possibly other factors.

#### 3.2.2. Other Mechanisms Conferring Resistance to Streptomycin

**Figure 3:**
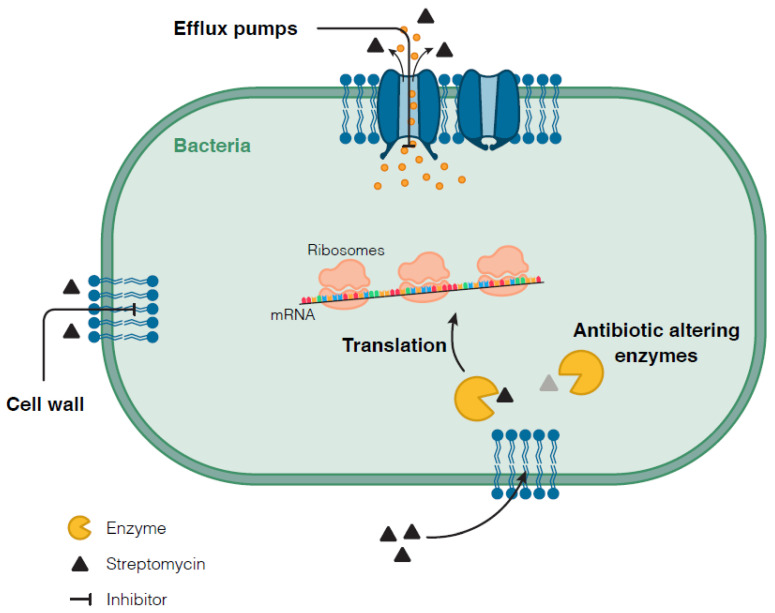
Schematic representation of general drug resistance mechanisms that could underly resistance to STR.

In addition to the resistant variants described on *rpsL*, *rrs*, and *gid*, the STR bactericidal effect can be avoided by other mechanisms collectively referred to as general DR mechanisms, some of which can be highly effective in *Mycobacterium*. Mutations affecting these mechanisms can decrease the susceptibility to different drugs (Figure 3) and are, thus, classified as unspecific resistant variants.

One of these mechanisms that is effective in preventing the activity of aminoglycosides implicates the phosphotransferase system. It was recently shown that the gene *Rv2004c*, from the *dosR* regulon, has mild phosphotransferase activity on STR. When analyzing the effect of STR phosphorylation, it was found that *Rv2004c* could mediate low levels of STR resistance [64]. Another STR resistance mechanism is likely related to *lipF* (Rv3487c), which encodes for a lipase with phospholipase C and carboxylesterase activities. The reduced expression of this gene is thought to contribute to the development of resistance to STR [65]. Moreover, the *M. tuberculosis* homocysteine synthase MetC (Rv3340), a key enzyme of the methionine synthesis pathway, was hypothesized to promote STR resistance. Indeed, overexpression of *metC* in an *M. smegmatis* mutant was shown to mediate STR resistance [66]. The transcriptional regulator *whiB7* was also involved in mechanisms of aminoglycoside resistance, including STR [67]. Mutations identified in the 5’ untranslated region of *whiB7* were associated with STR resistance, possibly due to the overexpression of Tap efflux pump (*Rv1258*), a mechanism strongly associated with tuberculous DR [67]. Interestingly, exposure to STR was shown to induce the expression of the *whiB7* gene [68,69]. In addition to Tap (Rv1258), the expression of *whiB7* can trigger the activation of other efflux mechanisms (like Rv01473) and other genes involved in the *M. tuberculosis* DR, such as *eis* (Rv2416c) and *erm*, potentially conferring resistance not only to STR but also to other antibiotics. In fact, cross-resistance to STR and Kanamycin was described in *M. tuberculosis* mutants containing resistant variants in the 5’ untranslated region of *whiB7* [67]. It was also highlighted that the overexpression of some cell wall and hypothetical proteins, like Rv1860, Rv1980c, Rv2140c, Rv1636, and Rv1926c, could be involved in STR resistance [70]. Recently a computational study evaluating a large collection of MDR *M. tuberculosis* from Peru associated the gene *ppsA* to STR resistance and other DRs. This association is probably linked to the contribution of *ppsA* (Rv2931) in the synthesis of PDIM (phthiocerol dimycocerosate) [51]. 

Collectively, the studies discussed above highlight that besides the resistant mutations found on *rpsL*, *rrs*, and *gid* genes, other mechanisms involving the cell wall permeability process and efflux pump proteins could also be implicated in the emergence and fixation of STR resistant *M. tuberculosis* strains, as proposed previously [55]. Therefore, to better detect and understand STR resistance, it will be relevant to fully uncover the STR “resistome” in *M. tuberculosis*. 

### 3.3. M. tuberculosis Mechanisms Associated with Tolerance to Streptomycin

A biological process that gained increasing attention in the last years in the context of TB treatment failure is antimicrobial tolerance. Phenotypic drug tolerance is likely caused by subpopulations of persistent cells, which are non-growing bacteria capable of inactivating important metabolic pathways to allow survival under stress conditions. Although the two definitions might be part of a continuous phenotypic spectrum, it is suggested that drug tolerance differs from antimicrobial resistance because drug-resistant bacteria can replicate during antimicrobial exposure while drug-tolerant bacteria stop growing [71]. Nonetheless, drug tolerance has the potential to be clinically relevant because of its possible association with relapse cases after the end of antimicrobial treatment [72,73,74]. It is important to highlight that prolonged TB treatments often trigger alterations of *M. tuberculosis* metabolic pathways and that these same pathways might mediate antimicrobial tolerance. Importantly, an in vitro study showed that the reference laboratory *M. tuberculosis* strain (H37Rv) developed tolerance to different antibiotics, including STR, through a mechanism requiring the sigma B factor [75]. Sigma factors are known to regulate *M. tuberculosis* slow-growing states and survival under stress conditions [76,77]. Furthermore, the Toxin Antitoxin (TA) systems were associated with these phenotypes in bacteria, including *M. tuberculosis* [78,79,80,81,82]. Interestingly, it was reported that exposure to STR results in the differential expression of many *M. tuberculosis* TA genes, including *vapC2*, *vapC32*, *mazE4*, *mazE5*, *mazE7*, *vapB38*, *vapC38*, and *vapB19* [83]. TA genes possibly contribute to increased survival in response to stress due to a shut-down of the translational mechanism, slow-down of metabolic processes, and promotion of a dormant state of the bacilli [83]. This fact could be particularly important as STR affects the translational capacity during protein synthesis. Another gene putatively involved in antimicrobial tolerance is *glpK*. In vivo studies suggested that clinical isolates containing *glpK* frameshift mutations in a 7C homopolymeric sequence could more rapidly switch to a reversible drug-tolerant state in the presence of different anti-TB drugs [84,85]. Recently, one of these mutations in the 7C homopolymeric sequence of *glpk* was also found in clinical isolates from a Portuguese cohort of pulmonary TB patients, which may be associated with tolerance to STR [23]. However, the contribution of *glpk* mutations to drug tolerance remains debatable [84,86,87,88,89], and further functional studies are needed to clarify its possible role. 

## 4. Streptomycin-Resistant Mutations and Fitness Costs

### 4.1. Does Streptomycin Predisposes M. tuberculosis to Other Drug Resistances?

The hypothesis that STR resistance can predispose to resistance to other drugs has been raised in different studies [19,37,38,90]. Research with *Escherichia coli* showed evidence pointing to a beneficial role in the combination of mutations associated with resistance to STR and RIF [91,92]. In these studies, it was shown that double-resistant STR and RIF *E. coli* can compensate for the fitness cost of drug resistance mutations faster than single-resistant bacteria [91,92]. Interestingly, attention has been called to the fact that the STR resistant bacterial populations are more likely to have mutations that compensate for fitness costs than RIF resistant ones [92]. This observation constitutes a potential problem as the emergence and fixation of resistant variants with low fitness cost could mitigate the cost of other drug-resistant mutations and consequently link with the acquisition of other DRs [93,94]. Indeed, studies based on the phylogenetic analysis of the whole genome sequence of clinical isolates have placed STR resistance as one of the early precursors in the order of acquisition of polyresistance in *M. tuberculosis* [19,36,37,38,39].

The fact that several studies show that, in TB, cross-resistance combining STR with other first and second-line antibiotics is common [11,34,67,95,96,97,98,99] could be due to the early introduction of STR in the history of antibiotic use and the current prevalence of specific STR mutations with low fitness costs. However, considering the reported studies gives strength to the hypothesis that isolates that become STR resistant could better compensate the cost of mutations conferring resistance to other antibiotics, eventually becoming more predisposed to polyresistance. Further studies are needed on this topic as the ability to forecast the evolutionary success of resistant mutants would be key to controlling the emergence and dissemination of antibiotic resistance [100].

### 4.2. Fitness of M. tuberculosis Lineages with Streptomycin-Resistant Mutations

It is conceivable that the impact of a specific resistance mutation in the fitness of an isolate can be influenced by its genomic context. In a cohort from a rural area in Vietnam, the transmission clustering of the L2 “Beijing genotype” was significantly more frequent if the infecting strain was resistant than if it was susceptible to STR, but the same was not observed among L1 strains. So, resistance to STR seems to have an advantageous fitness cost among the L2 “Beijing genotype” and a different fitness effect across genotypes [38]. A higher proportion of STR resistant mutations among the L4, when compared to L1, was also demonstrated in the other Vietnamese regions. In a national survey performed in China, it was found that LAM was the most transmitted sub-lineage among the L4 genotype and was strongly associated with STR resistance [101]. Yet, the fixation of STR resistance was shown to be higher for L2 “Beijing” than for L4 strains [37]. These works suggest that the fitness effect of STR resistance could vary among different *M. tuberculosis* lineages. However, the contribution of the type of DR mutation or compensatory mechanism is still elusive and might influence or modulate the fitness effect, ultimately affecting the development and clinical use of new and repurposed drugs [102]. 

Overall, the high frequency of STR resistance may result from the high prevalence of resistant mutations in STR target genes that impose low or no fitness costs for the bacteria, thus allowing an efficient transmission and persistence in the population. As an example, *M. tuberculosis* bacilli harboring Lys43Arg and Lys88Arg mutation in *rpsL* showed similar relative fitness compared with susceptible strains [103]. These facts probably explain why the substitutions Lys43Arg and Lys88Arg are frequently found in vivo while other variants on *rpsL* are less frequently found. In contrast, other polymorphisms in *rrs* and *rpsL* have been shown to reduce translation efficiency and consequently fitness of the pathogen [104,105]. To date, the fitness effect of *gid* resistant mutations has been poorly explored [105]. However, the strong association of *gid* resistant mutations with widely spread genotypes is also suggestive of a neutral or even advantageous impact on the fitness of the strains. An example of the high transmission of *gid* mutations conferring STR resistance is the presence of the *gid* polymorphisms Val100fs and Ala80Pro in different MDR outbreaks (denoted as MDR clusters M and Q1, respectively) [21,106]. Recently, we found that STR resistant clinical isolates with *gid* mutations were not affected in fitness and were transmitted in different inferred transmission clusters in the North of Portugal [23]. It is possible that compensatory mutations might play a role in the transmissibility and persistence of these STR resistant strains in the population, even in the absence of antibiotic pressure. It has been previously shown that the polymorphism Ile1106Thr in the *rpoB* and several others in *rpoA* can restore the fitness of STR resistant strains. Amino acid changes in *rpoB* and *rpoA* are also involved in the resistance and compensation of RIF resistance [104]. The report that mutations in *rpoA*/*B* can compensate the cost of both STR and RIF resistance can be seen as another example of the positive epistatic interaction between resistance to different drugs used to treat TB that is possibly relevant in the evolution towards MDR. This dual effect can probably explain the high frequency of STR resistance among MDR clinical isolates. Importantly, the existence of *M. tuberculosis* isolates with low fitness cost DR is worrisome as these bacteria can continue to evolve along with the transmission between hosts, potentially leading to higher resistance. In addition to *rpoA*/*B*, studies in other bacteria highlight *rpsD* and *rpsE* as potentially playing a role in the compensation of STR resistance costs [104,106,107]. Typically, the amino acid mutations in *rpsD* and *rpsE* were shown to occur after stress, affecting translation efficiency [108]. Interestingly, it was predicted *in silico* that *rpsE* can interact with *rpoB* [109]. The study also proposed that *rpsL*, one of the main targets of STR, is also involved in a possible association between the gene *rpsD* and the dormancy of RIF resistant “Beijing strains” [109]. It has been proposed *M. tuberculosis* uses this dormancy state to resist the antimicrobial effect. *Rv0516c*, *murA*, *cobL*, *cyp137* (Rv3685c), and *fadD34* were described as having direct epistasis with the polymorphisms *rpsL:88* conferring STR resistance on Ural *M. tuberculosis* sub-lineage. Similarly, *cfpA fadE36*, *lppP*, and PE-PGRS family protein *Rv0578* were linked with the mutation *rpsL:43.* These interactions were identified as prerequisites for the development of the resistance [110]. This fact shows that the fixation of STR resistance mutations and spread through the population might have benefited from different compensatory mechanisms. These mechanisms could be epistatic and potentially affect resistance to STR and other drugs.

## 5. Conclusions and Future Perspectives

STR is, nowadays, less used for TB therapy. However, resistance to this antibiotic is among the most common in different world regions. STR resistance seems to be one of the most persistent DRs in TB. The mechanisms that allowed for STR resistant bacteria to continue transmitting in the population, possibly since the first *M. tuberculosis* STR resistant strains emerged decades ago, remain elusive. *M. tuberculosis* might have evolved different mechanisms that promoted the fixation of STR resistance even without strong selective pressure. The hypothesis that the STR resistant strains that are now circulating are highly transmissible and possibly more prone to acquire other DRs is worrisome and should not be neglected. In this context, uncovering STR resistant-conferring mutations associated with low fitness cost and high frequency and transmissibility, especially among MDR *M. tuberculosis*, is an important research question. Similarly, investigating fitness compensatory mutations among STR resistant strains and how they may articulate with MDR development is a matter of interest and a relevant topic for future research. Although many studies have explored the role of STR resistance on the fitness of different bacteria, unfortunately, in the case of *M. tuberculosis* there is a limited body of evidence addressing this issue [48,92,110,111,112]. In recent work with a Portuguese cohort of pulmonary TB, it was found that the STR resistant clinical isolates were indistinguishable from susceptible isolates in what concerns in vitro growth, a commonly used surrogate for fitness [23,113,114]. Importantly, this work has identified isolates evolving at the transmission cluster level, from low to high streptomycin resistance levels, without a significate fitness cost [23]. In future studies, combining whole-genome analysis and experimental genetics might provide a more complete picture of the effect of STR resistance on the transmission fitness of the bacilli, and to which extends this specific DR influence the emergence of MDR [104,106]. In addition, computational and phylogenetic methods to infer transmission fitness are advancing and could be used to investigate the dynamics of new cases per time caused by a patient infected with an STR resistant strain or specific mutation(s) compared to a patient with a drug-susceptible strain [111]. In line with previous studies [115,116,117], competition assays of *M. tuberculosis* isolates with and without different STR resistant mutations could also be relevant tools to study in vitro fitness surrogates. Studying the rates of acquisition and fitness costs of different STR resistance mutations in DR clinical isolates is also considered relevant. These studies could identify compensatory mutations affecting the growth, transmissibility, and propensity of DR. 

Overall, the state-of-the-art in this topic suggests that STR resistance, which has not been perceived as a major clinical threat, should be better studied. It would not be surprising if STR-resistant bacteria had a key role in the ongoing and emerging problem of antimicrobial-resistant TB. STR resistance could be a relevant model to understand how DR resistance is maintained in different populations of *M. tuberculosis* and to investigate the recent evolution of transmission and how it affects DR resistance level, fitness, and potentially predisposes to MDR.

## Figures and Tables

**Table 1:** List of mutations in *rpsL*, *rrs*, and *gid* associated in the literature with STR resistance.

Gene Name	Polymorphism (Nucleotide or Amino Acid Change)	Suggested Reference
*rpsL*	Lys88Arg, Lys43Arg	[59]
	Arg86Pro Arg86Tyr, Arg9His, Gly84Val, Lys43Thr, Lys51Asn, Lys88Gln, Lys88Met, Thr40Ile, Thr41Ser, Val52Gly, Val87Leu, Val93Met	[60]
	Arg86Gly	[30]
	Gly118Asp	[55]
*rrs*	190G/A, 277G/C, 427G/C, 462C/T, 513C/T, 514A/C, 516C/T, 517C/T, 628G/C, 799C/T888G/A, 905C/A, 905C/G, 906A/G, 907A/C, 907A/T, 908A/G	[60]
	644A/G	[55]
*gid*	102del, 103_104insG, 107del, 115del, 136del, 157del, 225_226insT, 294_295insAC, 297_298insA, 326del, 351del352_353insG, 366_367del, 386del, 390del, 400_401insT, 446_447insA, 450del, 452del, 455del, 471del, 519_520insA, 554_555insG, 559_572del, 58_59insT, 601del	[61]
	Leu108Arg, Leu35fs *, Ala200Glu, Cys191Arg, Gly73Glu, Cys191Arg, Gly73Glu, Leu50Arg, Glu60fs *, Arg39fs *, Arg118fs *, Arg217Gly, Leu94Pro, Asp67Gly, Pro84Leu, Gly73Ala, Leu145Phe, Val77Gly, Val135fs *, Ser149Arg, Leu90Phe, Gly214Ala, Ala119Thr; Ala19Ser, Arg158Leu, Val66Leu, Arg137Gln, Ala134Glu, Ala138Val	[61]
	259C/T	[59]
	Pro75Thr	
	Arg47Gln, Pro84Ser, Met104dup, Gly117Arg, Lys163Asn, Ile11Thr, Cys119Trp, Cys191Phe, Ser70Arg, Ala141Glu	[23]
	Ala80Pro, Tyr195His	[21]
	His168Tyr, Gly208Val	[30]
	Ala19Gly	[55]
	Met218Val	[55]
	Val110fs	[62]
	Gly13Arg, Leu101Phe	[63]
	243insGC, 112delC, 254delA, 347delG, 372delG	[55]

* Frameshift mutation (fs).
